# Understanding the Central Nervous System Symptoms of Rotavirus: A Qualitative Review

**DOI:** 10.3390/v13040658

**Published:** 2021-04-11

**Authors:** Arash Hellysaz, Marie Hagbom

**Affiliations:** Division of Molecular Medicine and Virology, Department of Biomedical and Clinical Sciences, Linköping University, 58183 Linköping, Sweden; arash.hellysaz@liu.se

**Keywords:** rotavirus, disease symptoms, central nervous system, enteric nervous system, gut–brain communication, gastroenteritis

## Abstract

This qualitative review on rotavirus infection and its complications in the central nervous system (CNS) aims to understand the gut–brain mechanisms that give rise to CNS driven symptoms such as vomiting, fever, feelings of sickness, convulsions, encephalitis, and encephalopathy. There is substantial evidence to indicate the involvement of the gut–brain axis in symptoms such as vomiting and diarrhea. The underlying mechanisms are, however, not rotavirus specific, they represent evolutionarily conserved survival mechanisms for protection against pathogen entry and invasion. The reviewed studies show that rotavirus can exert effects on the CNS trough nervous gut–brain communication, via the release of mediators, such as the rotavirus enterotoxin NSP4, which stimulates neighboring enterochromaffin cells in the intestine to release serotonin and activate both enteric neurons and vagal afferents to the brain. Another route to CNS effects is presented through systemic spread via lymphatic pathways, and there are indications that rotavirus RNA can, in some cases where the blood brain barrier is weakened, enter the brain and have direct CNS effects. CNS effects can also be induced indirectly as a consequence of systemic elevation of toxins, cytokines, and/or other messenger molecules. Nevertheless, there is still no definitive or consistent evidence for the underlying mechanisms of rotavirus-induced CNS complications and more in-depth studies are required in the future.

## 1. Introduction

In children under the age of five, rotavirus is the most common causative agent of severely acute gastroenteritis worldwide [1,2]. The major symptoms of rotavirus infection of the small intestine are diarrhea and vomiting, which can cause rapid dehydration in young children and even lead to organ failure and death. Although rotavirus infects the small intestine, most of its symptoms, such as vomiting, sickness feeling, fever, loss of appetite, and fatigue, indicate signaling via the central nervous system (CNS). In fact, brain–gut communication in rotavirus infection [3,4] is suggested to play an important role in its major symptoms [5,6]. Additionally, there is growing evidence of CNS complications, such as convulsions, encephalitis, and encephalopathy, that may be associated with rotavirus infections [7,8,9]. Yet, definitive and consistent evidence of the underlying mechanisms are largely lacking, making it difficult to review and explain how the pathogen causes these various CNS associated symptoms. This review therefore aims to create a better understanding of CNS complications caused by rotavirus infection, by exploring published literature on rotavirus infections and CNS symptoms.

## 2. Histopathological Mechanisms of Rotavirus Infection

Rotavirus infects enterocytes in the small intestine, where the virus exhibits tropism towards mature enterocytes and only infects the middle and top portions of the villi [10]. Rotavirus also infects sensory enterochromaffin (EC) cells [11,12], but these cells are few, less than 1% of the intestinal epithelium [13]. Histopathological analysis has demonstrated vacuolization of the enterocytes and shortening and blunting of villi in the rotavirus-infected intestine [14,15].

## 3. Brain–Gut Communication in Rotavirus Infection

The cellular changes observed in the rotavirus-infected intestine appear before the onset of symptoms and resolve before viral clearance; this indicates that the onset of diarrhea and vomiting might be caused by mechanisms other than direct pathological effects [16]. Several hypotheses for the mechanism by which rotavirus could induce diarrhea have been proposed, such as villus ischemia, reduced absorptive capacity of the enterocytes, effects of the rotavirus enterotoxin non-structural protein 4 (NSP4), and activation of the enteric nervous system (ENS) [17].

During rotavirus replication in enterocytes, the enterotoxin NSP4 is produced and released. NSP4 stimulates neighboring EC cells to release serotonin and activates nerves within the ENS as well as vagal afferents to the brain [11], causing diarrhea and vomiting [18,19,20,21,22]. Serotonin is a mediator involved in intestinal motility [19,23,24,25], secretion [21,22,26], pain sensation [27,28,29], regulation of inflammation [30,31], and activation of the vagus nerve will propagate the signal to the brain and elicit, e.g., vomiting [20,32]. These findings indicate the important role of the mediators, such as NSP4 and serotonin, but also the gut–brain axis, to explain rotavirus symptom mechanisms.

## 4. Role of the ENS in Rotavirus Infection

The first study to investigate the role of the ENS in rotavirus disease was published by Lundgren et al. two decades ago [33], who by applying drugs that inhibit ENS functions on an in vivo mice model, confirmed that rotavirus induces intestinal fluid and electrolyte secretion by ENS activation. The ENS and intestinal epithelium cells are in close contact with the vagal nerves [34]. About 80–90% of these vagal nerves are afferents that project from the gut and send sensory information to the brain. The remaining 10–20% of the vagal nerves are efferents projecting to the periphery to modulate various effects [35]. From an evolutionary point of view, symptoms of rotavirus infection are not specific to the pathogens *per se* but are rather generated as general protective mechanisms to combat infections and eliminate pathogens and their toxins [36].

## 5. CNS Complications in Rotavirus Infection

The first study to connect rotavirus infection and the CNS was published by Salmi et al. in 1978 [37] presenting two clinical cases of children with rotavirus gastroenteritis, where one had developed a fatal Reye’s syndrome and the other one encephalitis with a slow recovery. Since then, rotavirus-induced CNS involvement has, in young children, been associated with seizures/convulsions [9,38,39,40], meningitis [41], encephalitis [37,42,43], hemorrhagic shock [44], Guillain–Barré syndrome [45,46,47], cerebellitis [48,49,50,51,52,53,54], and encephalopathy [37,55,56]. The actual frequency of these CNS symptoms remains unclear but has been estimated to be between 2% and 6% [7,57,58,59]. Nevertheless, the mechanisms by which rotavirus causes the CNS effects are still under debate.

Rotavirus antigen (antigenemia) and free unenclosed genomic RNA are commonly (65% of cases) detected in the serum of children with rotavirus diarrhea [60], and viremia have also been reported [60,61,62]. Case reports from immunodeficient children with rotavirus gastroenteritis have described the detection of rotavirus RNA in organs such as the liver, kidneys, and the CNS [63,64]. Rotavirus antigen has also been detected in cerebrospinal fluid (CSF) of children with mild or severe convulsions, or encephalitis [39,41,42,43,55,65,66,67,68]. Additionally, nucleotide sequencing revealed an identical rotavirus strain in the stool and CSF samples of a patient with concurrent rotavirus gastroenteritis and signs of CNS complications; a finding that implies that rotavirus was able to spread from the gastrointestinal tract to the CNS, where it probably played a role in the onset of neurological disease [69].

Studies using diffusion-weighted imaging also suggest a connection between rotavirus infection and white matter injury (WMI), however, without direct invasion of the CNS [70,71]. Cerebral WMI is recognized as the most common form of injury in the developing brain of neonates and is also thought to be associated with seizures. Rotavirus, enterovirus, and parechovirus have been found in newborns with WMI [70], but in contrast to enterovirus and parechovirus, rotavirus was not found in the CSF, and CSF pleocytosis was not detected in rotavirus-infected children with WMI. Moreover, fever and rashes, as responses to inflammation, are rare in neonates suffering from WMI and rotavirus infection. Activation of brain microglia, excitotoxicity, and free radical attack has been suggested to be the downstream event to systemic infection, and inflammation leading to WMI [72]. However, since rotavirus was not detected in CSF [40,73,74] and inflammatory cells were not detected in the CSF and the brain [73], the mechanism for how rotavirus may cause WMI in neonates remain unclear.

## 6. Pathophysiology of CNS Complications

The rotavirus enterotoxin NSP4 is considered to be one of the mediators causing neurological complications [75]. Rotavirus has been shown to infect neuronal cells in vitro, and viral proteins has been identified in both axons and dendrites [76]. These findings indicate that rotavirus might be able to invade the CNS and have a direct effect or replicate and induce neurotransmitter dysregulation. This hypothesis is, however, somewhat controversial, since rotavirus RNA is not always detected in the CSF. Another hypothesis is that mediators in circulation, such as prostaglandins [77], cytokines [49,78,79], reactive oxygen species [80], rotavirus RNA, or NSP4, may act as secondary messengers and indirectly induce CNS effects. Additionally, increased levels of excitatory amino acids have also been found in the CSF, which may induce neurological disorders and are possibly related to the severity of the disorder [81].

Following oral inoculation of murine rotavirus in mice, rotavirus-specific proteins have been detected in macrophages and B-cells in gut-associated lymphoid tissue [82], providing the lymphatic system as yet another route for extra-intestinal spread.

The blood–brain barrier (BBB) consists of highly selective semipermeable border of endothelial cells that, under normal conditions, prevents direct entry of solubles into the CNS and thereby protects the brain from injury [83]. However, several regions of the CNS, such as the area postrema (AP), subfornical organ, pineal gland, and median eminence of the hypothalamus, collectively known as the circumventricular organs (CVOs), have fenestrated capillaries that lack conventional BBB properties and enable some vascular permeability. These regions could provide a doorway, from where blood-borne rotavirus could enter the brain. During disease, several molecular factors, including inflammatory cytokines (TNF-α, IL-1, and IL-6) and reactive oxygen species, can cause BBB dysfunction [83]. These mediators have also been shown to be increased during rotavirus infection [84,85]. Rotavirus genomes have been discussed to pass the BBB with increased vascular permeability during convulsions or through infection of neuronal cells and release into the CSF [86]. However, the causality of these observations remains obscure. As rotavirus is not always found in the CSF, it has been suggested that it either only exists for a very short time in the CSF or it is present in concentrations below detection limit [84].

**Figure 1:**
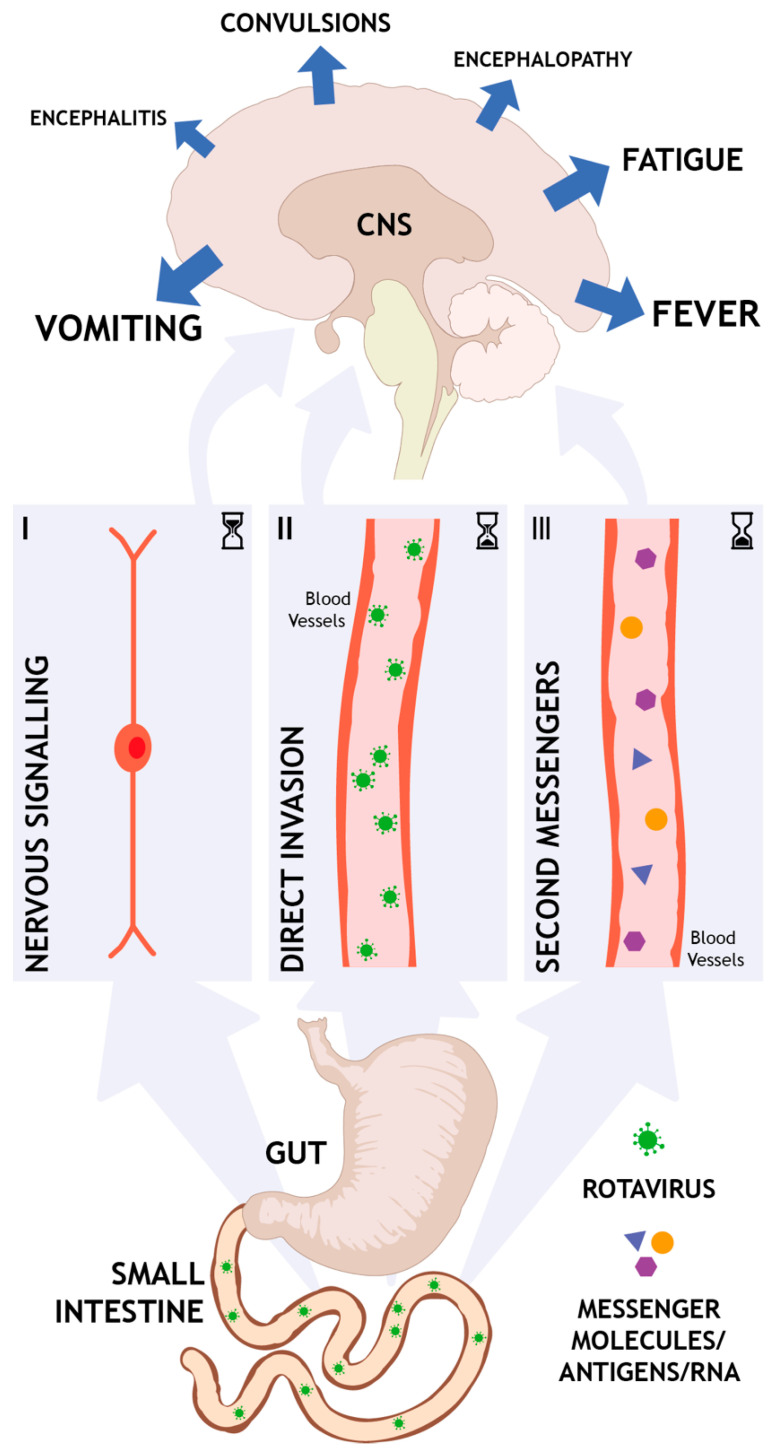
Schematic representation of possible routes by which rotavirus infection can affect the brain to give rise to central nervous system (CNS)-associated symptoms. From very early on, infection-induced released mediators like rotavirus enterotoxin nonstructural protein 4 and serotonin can activate nerves (**I**) that propagate the signal to the brain. In hosts suffering from malnutrition, immunosuppression, or immunodeficiency, virus/-antigen/-RNA may enter the bloodstream and/or the lymphatic system, and together with dysfunction in the blood brain barrier there is a possibility that they directly enter the brain (**II**) to cause less prevalent symptoms like encephalitis or encephalopathy. However, evidence that rotavirus enters the brain is lacking. Finally, infection-induced systemic elevation of rotavirus-antigens or -RNA, toxins, cytokines, and/or other messenger molecules may indirectly (**III**) affect the brain. Routes are not exclusive and could overlap or occur at different timepoint in the same host. Hour glasses represent time lapse post infection. Less common symptoms represented by smaller arrows and text.

Several mechanisms have been proposed to explain the CNS complications of rotavirus infection, see Figure 1. However, conclusive evidence linking rotavirus and the CNS are largely lacking and many aspects of the pathophysiology remain elusive.

## 7. Conclusions

Symptoms such as nausea, vomiting, pain, and sickness feeling are commonly associated with rotavirus gastroenteritis and likely to involve the CNS. Currently reviewed literatures strengthen this view and clearly indicate the CNS in the underlying mechanisms of rotavirus symptoms. The literature provide evidence for three different routes by which rotavirus infection could activate the brain and give rise to various CNS symptoms (see Figure 1).

1. Nervous route. At the site of infection in the intestines, released mediators such as NSP4 and/or serotonin can activate the ENS and vagal afferents that, through nervous communication, signal to the brain. This nervous pathway is direct, fast, can occur early in the course of the disease, and is not interrupted by defense mechanisms like the BBB.
2. Direct invasion. In a host that suffers from malnutrition or immunodeficiency and exhibit e.g., dysfunction in the BBB, virus can potentially enter the brain. The virus can also enter the lymphatic system, from where it is spread to other organs, including the CNS. This route obviously requires a weakened host and can only occur later in the course of the disease when the virus has already replicated several rounds and virions are present in high titers. However, the causality of direct invasion remains obscure since rotavirus cannot always be found in CSF.
3. Second messengers. Systemic elevation of toxins, cytokines, and/or other messengers can indirectly induce CNS effects. This kind of CNS response is per definition occurring later in the course of the disease and is likely part of a coordinated immune response.

Naturally, these routes are not exclusive and could all occur in the same host.

The broad function of the CNS is to maintain homeostasis by interpreting sensory information and creating motor responses. Protecting the host from pathogenic invasions is part of this broad function, and there is an evolutionary drive for the CNS to respond to pathogens accordingly. For example, fever is evolved as an organized strategy to combat viral and bacterial infections [87]. Similarly, vomiting is another strategy for the host to rapidly get rid of infected and/or toxic food. Food intake is a risky behavior that exposes the host to viral and bacterial pathogens and toxins [5]. The ability to rapidly, via gut–brain communication, identify a threat and activate diarrhea and vomiting, and subsequently to flush out toxins and pathogens and reduce exposure and risk for uptake, provides an evolutionary advantage that is not specific to rotavirus infection.

Invading pathogens, other exogenous factors, or factors produced by cells within the brain can cause neuro-inflammation, which is orchestrated through the activation of microglia, astrocytes, neurons, endothelial cells, and pericytes [88]. On activation, glial cells and neurons closely interact with each other and communicate to regulate barrier properties and inflammatory responses. In fact, the endothelium–microglia interface constitutes the first line of defense of the CNS against injury [88]. The permeability of the BBB is increased in some children during rotavirus infection. Thus, in children exhibiting CNS effects such as convulsions, encephalitis, and encephalopathy, the virus or viral antigen may have entered the nerves or bloodstream and caused direct or indirect effects on the CNS [89].

The vagal nerves, which represent the gut–brain connection, have been shown to regulate permeability [90] and inflammation [91,92,93,94]. Similarly, enteric glia cells in the gut have been shown to regulate intestinal inflammation and permeability [95,96,97], and it is believed that microglia cells in the brain may have similar functions [98]. Therefore, based on these findings, the CNS effects in young children could be due to a low vagus nerve tone or by mechanisms that weaken barrier function.

Despite the multitude of evidence for the involvement of the CNS in rotavirus pathophysiology, defined pathways and the mechanistic explanation of most symptoms are still absent. This lack of causal links is partly due to technical limitations, making it difficult to detect and to conduct the required holistic investigations in two large organs at great distance afar. However, emerging new techniques such as 3D imaging of whole organs at cell resolution and co-cultivation of enteroids or organoids with enteric glia cells and neurons provide new opportunities to investigate and connect the components that drive gut–brain communication during viral infections. Such knowledge will provide a better understanding of rotavirus pathophysiology and enable the development of more specific and efficient therapies in the future.

## Data Availability

No new data were created or analyzed in this study. Data sharing is not applicable in this article.
